# Normalizing the political economy of improving health

**DOI:** 10.2471/BLT.21.286629

**Published:** 2022-03-03

**Authors:** Susan P Sparkes, Paola Abril Campos Rivera, Hyobum Jang, Robert Marten, Dheepa Rajan, Alastair Robb, Zubin Cyrus Shroff

**Affiliations:** a Health Systems Governance and Financing, World Health Organization, Avenue Appia 20, 1211 Geneva 27, Switzerland.; b Delivery for Impact, World Health Organization, Geneva, Switzerland.; c Country Strategy and Support, World Health Organization, Geneva, Switzerland.; d Alliance for Health Policy and Systems Research, World Health Organization, Geneva, Switzerland.; e Global Malaria Programme, World Health Organization, Geneva, Switzerland.

## Abstract

**Problem:**

Political economy factors are important in determining the adoption and implementation of health policies. Yet these factors are often overlooked in the development of policies that have the potential to influence health.

**Approach:**

Political economy analysis provides a way to take into consideration political and social realities, whether at the community, subnational, national, regional or global levels. We aim to demonstrate the value of political economy analysis and to promote its wider use in technical programmes of work.

**Local setting:**

We provide examples from across a range of World Health Organization areas of work, including participatory governance, health financing, health taxes, malaria prevention and control, capacity-building and direct country support.

**Relevant changes:**

Existing examples of how political economy analysis can be incorporated into technical support demonstrate the variability of this analytical approach, as well as its potential to support policy progress. Applying political economy analysis within the specified programmes of work has enabled more contextually relevant technical support to enhance the likelihood of advancing countries’ health-related objectives.

**Lessons learnt:**

Embedding political economy into technical work has many benefits, including: enhancing voice and participation in health policies; supporting the adoption and implementation feasibility of technically sound policies; and building capacity to incorporate and understand political factors that influence health-related priorities.

## Résumé

**Problème:**

Les facteurs liés à l'économie politique jouent un rôle crucial dans l'adoption et la mise en œuvre de mesures sanitaires. Pourtant, ces facteurs sont souvent négligés lors de l'élaboration de politiques susceptibles d'avoir un impact sur la santé.

**Approche:**

Analyser l'économie politique représente un moyen de tenir compte des réalités politiques et sociales au niveau communautaire, infranational, national, régional ou international. Nous voulons démontrer l'importance de l'analyse de l'économie politique et promouvoir son usage dans les programmes techniques de travail.

**Environnement local:**

Nous donnons des exemples issus d'une série de domaines traités par l'Organisation mondiale de la Santé, parmi lesquels la gouvernance participative, le financement de la santé, les taxes sanitaires, la prévention et la lutte contre la malaria, le renforcement des capacités et l'appui direct aux pays.

**Changements significatifs:**

Les exemples portant sur l'intégration de l'analyse de l'économie politique dans l’appui technique illustrent la variabilité de cette approche analytique, mais aussi sa potentielle contribution aux progrès politiques. Appliquer une analyse de l'économie politique à des programmes de travail spécifiques a permis de fournir un soutien technique adapté au contexte, augmentant ainsi les chances d'avancement des pays vers la réalisation des objectifs en matière de santé.

**Leçons tirées:**

Incorporer l'économie politique dans le travail technique comporte de nombreux avantages: amélioration de la représentation et de la participation dans le cadre des mesures sanitaires; meilleures possibilités d'adoption et de mise en œuvre de politiques solides sur le plan technique; et enfin, renforcement des capacités afin de comprendre et d'inclure les facteurs politiques qui ont une influence sur les priorités relatives à la santé.

## Resumen

**Situación:**

Los factores de economía política son importantes para determinar la adopción y aplicación de las políticas sanitarias. Sin embargo, se suelen ignorar estos factores cuando se elaboran políticas que pueden influir en la salud.

**Enfoque:**

El análisis de economía política permite tener en cuenta las realidades políticas y sociales, ya sea a nivel local, subnacional, nacional, regional o mundial. El objetivo de este proyecto es demostrar el valor del análisis de la economía política y promover su uso generalizado en los programas técnicos de trabajo.

**Marco regional:**

Se ofrecen ejemplos de diversas áreas de trabajo de la Organización Mundial de la Salud, como la gobernanza participativa, la financiación sanitaria, los impuestos sanitarios, la prevención y la contención del paludismo, la creación de capacidades y el apoyo directo a los países.

**Cambios importantes:**

Los ejemplos existentes de cómo se puede incorporar el análisis de economía política al apoyo técnico demuestran la variabilidad de este enfoque analítico, así como su potencial para apoyar el progreso de las políticas. La aplicación del análisis de la economía política en los programas de trabajo especificados ha permitido que el apoyo técnico sea más pertinente según el contexto para aumentar la probabilidad de avanzar en los objetivos sanitarios de los países.

**Lecciones aprendidas:**

Integrar la economía política en el trabajo técnico tiene muchos beneficios, entre los que se incluyen: potenciar las opiniones y la participación en las políticas sanitarias; apoyar la adopción y la viabilidad de la aplicación de políticas técnicamente sólidas; y crear capacidad para incorporar y comprender los factores políticos que influyen en las prioridades sanitarias.

## ملخص

**المشكلة:**

تعد عوامل الاقتصاد السياسي ذات أهمية في تقرير اعتماد السياسات الصحية وتنفيذها. إلا أنه غالبًا ما يتم تخطي هذه العوامل عند تطوير السياسات التي لديها القدرة على التأثير على الصحة.

**الأسلوب:**

يوفر تحليل الاقتصاد السياسي وسيلة لوضع الحقائق السياسية والاجتماعية في الاعتبار، سواء على مستوى المجتمع، أو المستوى دون الوطني، أو الوطني، أو الإقليمي، أو العالمي. نحن نهدف إلى إظهار قيمة تحليل الاقتصاد السياسي، وتعزيز استخدامه على نطاق أوسع في البرامج الفنية للعمل.

**المواقع المحلية:**

نحن نقدم أمثلة عبر مجموعة من مجالات عمل منظمة الصحة العالمية، بما في ذلك الحوكمة المشتركة، والتمويل الصحي، والضرائب الصحية، والوقاية من الملاريا ومكافحتها، وبناء القدرات، والدعم المباشر على مستوى الدولة.

**التغيّرات ذات الصلة:**

إن الأمثلة القائمة لكيفية دمج تحليل الاقتصاد السياسي في الدعم الفني، توضح تنوع هذا الأسلوب التحليلي، فضلاً عن قدرته على دعم تقدم السياسة. إن تطبيق تحليل الاقتصاد السياسي ضمن برامج العمل المحددة قد أتاح المزيد من الدعم الفني ذي الصلة بالسياق، لتعزيز احتمالية الارتقاء بالأهداف المتعلقة بالصحة في الدول.

**الدروس المستفادة:**

إن دمج الاقتصاد السياسي في العمل الفني له العديد من الفوائد، بما في ذلك: تعزيز الرأي والمشاركة في السياسات الصحية؛ ودعم اعتماد سياسات سليمة من الناحية الفنية وجدوى تنفيذها؛ وبناء القدرات لدمج العوامل السياسية التي تؤثر على الأولويات المتعلقة بالصحة، وفهمها.

## 摘要

**问题:**

政治经济因素在决定采纳和实施卫生政策方面发挥重要作用。然而，在制定可能影响卫生的政策时，这些因素往往被忽视。

**方法:**

政治经济学分析提供了一种考虑政治和社会现实的方法，无论是在社区、地方、国家、地区还是全球层面。我们的目的在于说明政治经济学分析的价值，并促进其在技术性工作计划中更广泛的使用。

**当地状况:**

我们提供一系列来自世卫组织工作领域中的示例，包括参与式治理、卫生筹资、医疗税、疟疾预防和控制、能力建设和直接国家支持。

**相关变化:**

有关如何将政治经济学分析纳入技术支持的现有示例表明该分析方法的可变性，以及该方法在支持政策进展方面的潜力。在特定工作计划中应用政治经济学分析，可以提供更加因地制宜的技术支持，以促进实现国家卫生相关目标的可能性。

**经验教训:**

将政治经济学融入技术工作有许多益处，包括：增强对卫生政策的发言权和参与度；支持采用和实施技术完备政策的可行性；建设能力以纳入和理解影响卫生相关优先事项的政治因素。

## Резюме

**Проблема:**

Факторы политической экономии играют важную роль в определении принятия и осуществления политики в области охраны и укрепления здоровья. Однако эти факторы часто упускаются из виду при разработке политики, которая способна повлиять на охрану здоровья.

**Подход:**

Политэкономический анализ позволяет учитывать политические и социальные реалии как на уровне сообщества, так и на субнациональном, национальном, региональном или глобальном уровне. Авторы ставят перед собой цель продемонстрировать ценность политэкономического анализа и способствовать его более широкому использованию в технических программах работы.

**Местные условия:**

В статье приводятся примеры из различных областей деятельности Всемирной организации здравоохранения, включая управление на основе широкого участия, финансирование здравоохранения, налоги на здравоохранение, профилактику малярии и борьбу с ней, наращивание потенциала и прямую поддержку стран.

**Осуществленные перемены:**

Существующие примеры того, как политэкономический анализ может быть включен в техническую поддержку, свидетельствуют об изменчивости этого аналитического подхода, а также о его потенциале для поддержки прогресса в политике. Применение политэкономического анализа в рамках указанных программ работы позволило получить техническую поддержку, более соответствующую контексту, для повышения вероятности достижения странами целей, связанных со здравоохранением.

**Выводы:**

Внедрение политэкономии в техническую работу имеет ряд преимуществ, в том числе повышение роли и участия в политике здравоохранения, поддержка принятия и реализации технически обоснованной политики, наращивание потенциала для учета и понимания политических факторов, оказывающих влияние на приоритеты в области здравоохранения.

## Introduction

As the coronavirus disease 2019 pandemic has demonstrated, policies that impact health and well-being are not only influenced by scientific evidence, but also by the political views and underlying values of policy-makers and societies.^1^ The need to make policy decisions in the face of uncertainties and incomplete information complicates these influences. Given the inextricable link between politics and public health, approaches are needed to systematically assess, analyse and incorporate the two factors as part of efforts to improve health.

Throughout this article we aim to demonstrate the value of political economy analysis in technical programmes of work to improve health and well-being. Analysing political economy provides a way to respond to and take into consideration the political, economic and social forces that impact health and related policies, whether at the community, subnational, national, regional or global levels. 

## Approach

Politics has been described as “who gets what, when, and how.”^2^ If we apply this concept to health then any number of inputs, variables and factors influence the distribution of health resources and services. As technical advisors working for the World Health Organization (WHO) we are often faced with considerations around the political dimensions of health and related reforms. Political economy analysis (Box 1) grounds our technical work in a structured assessment of the politics and economics of a given context.^3^ By applying this method, we recognize the role of politics, social values, economics and institutions in shaping policy decisions that impact health.

Box 1 **Definition of political economy analysis**

Political economy analysis is the analysis of the interaction of political and economic processes in a society: the distribution of power and wealth between different groups and individuals, and the processes that create, sustain and transform these relationships over time.

Source: Department for International Development, 2009.

We outline some recent examples of how we have incorporated political economy analysis into the many aspects of WHO’s technical work. The examples, which are not intended to be a complete or representative selection of our work, can be grouped into three themes. The first theme is efforts around enhancing people’s voice and participation in policy- and decision-making. Participatory governance is core to the values embedded within universal health coverage (UHC), including accountability, inclusivity, responsiveness and people-centredness. How these participatory approaches are established and managed is inherently political and directly impacts policy. However, countries’ capacity to convene different interest groups and to mediate and deal with conflicts of interests and power imbalances within a participatory programme is often weak. 

The second theme is making it more feasible for technically sound policies to be adopted and implemented. This theme includes health financing reforms that promote progress towards UHC. These reforms are highly political through their impact on the distribution of entitlements, responsibilities and resources across the health sector and beyond. Another policy area is health taxes, which are a key tool to generate public resources and promote improved public health.^4^ Yet emerging evidence suggests that how these taxes are developed and framed often determines their ability to achieve the intended revenue and health objectives.^5^^,^^6^ Finally, although gains in health can be made through technical solutions, the threats to durable progress are often rooted in the social, political and economic determinants of health.

The third theme is capacity-building to support Member States to embed political economy analysis into policy processes. In addition to incorporating political economy analysis within specific technical areas of work, there are efforts underway within WHO to systemize and embed these approaches into our overall support mechanisms to countries. By incorporating political economy analysis and approaches into strategic policy discussions, WHO country offices can take account of country contexts, improve technical advice related to health-system transformation, and link country partners with related global health initiatives. Beyond direct support to Member States, making political economy analysis a routine part of efforts to improve health requires adequate capacities within all countries, beyond WHO country offices, to carry out such analysis. 

## Local setting

We provide specific examples from across a range of WHO programmes of work that explicitly incorporate political economy analysis. The examples cover participatory governance, health financing, health taxes, combatting malaria, capacity-building and direct country support. We chose the examples to illustrate the wide range of ways to apply political economy analysis to work aimed at improving health and well-being for all.

## Relevant changes

Existing examples of how political economy analysis can be incorporated into technical support demonstrate the variability of this analytical approach, as well as its potential to support policy progress. This analytical approach helps us to adapt technical work to each individual context to support advances in health-related objectives that are feasible to implement.

### Participatory governance

In 2021, WHO published the *Handbook on social participation for universal health coverage*.^7^ Developed in collaboration with civil society groups, the handbook aims to improve participatory approaches towards UHC, to provide best practice guidance, and to gather and generate evidence. Acknowledging inherently unequal power relationships among individuals and groups in participatory governance, the handbook describes a process which guides participant selection and representativeness; highlights facilitation techniques to raise the voices of citizens with less influence and power; and recommends explicitly identifying groups within society that are not participating.

### Health financing reform

Given that many health financing reforms fail because of political constraints, WHO’s health financing team launched a programme of work on the political economy of health financing reform in 2019.^8^ The programme brings together the technical and the political aspects of health financing into a coherent process, rather than two separate streams that might diverge at some point. Building on countries’ experiences and expertise, our analysis underscores the value of explicitly incorporating stakeholder interests and relative power into health financing reform strategies.^9^

### Health taxes 

Political challenges in implementing health taxes can limit their usefulness. Indeed, more rigorous exploration of political economy factors and an understanding that how health taxes are framed can shape their implementation is needed.^10^ To help rectify this, in 2021 the Alliance for Health Policy and Systems Research, a WHO-hosted partnership, launched a programme of work to identify and build the capacity of local health researchers, as a way to support policy-makers in communicating and advancing health taxes. 

### Political commitment

Despite impressive achievements in reducing malaria morbidity and mortality over the past 20 years,^11^ there has been limited progress since 2015. The High Burden to High Impact approach, launched by WHO and the Roll Back Malaria Partnership to End Malaria in 2018,^12^ is a country-led approach to get back on track to achieve the goals of WHO’s Global Technical Strategy for Malaria 2016–2030. Increasing political commitment to combat malaria is one of the four response elements of the approach. By analysing their political context, countries with a high burden of malaria are developing locally appropriate strategies that are embedded in, and supported through, strong and equitable health systems. 

### Direct country support

WHO’s country offices work closely with health ministries to develop country cooperation strategies, through which WHO provides technical advisory support, as well as bringing together all sectors and partners around collaborative, consensus-driven approaches at country level. 

Implementing strategic documents often entails building consensus and managing conflict across a range of individuals and institutions.^13^ The WHO Delivery for Impact team and knowledge hub formed in 2020 builds capacity and supports Member States in the process of implementing health policy towards measurable improvements in health. In explicitly recognizing the power dynamics involved in policy implementation, WHO’s approach includes guidance on incorporating continual stakeholder analysis: a process which assesses the power and position of key individuals or groups in both health and non-health sectors. The process allows decision-makers and others working on health reform to anticipate implementation challenges stemming from the different interests, opinions and positions of power among stakeholders and to develop strategies to mitigate the risk of failure.

### Capacity-building

To build capacity to support Member States in using political economy analysis, the Alliance for Health Policy and Systems Research has developed the health policy analysis fellowship programme.^14^ In 2017, in collaboration with the University of Cape Town, South Africa, the programme (now in its second round) has supported over 20 doctoral candidates in low- and middle-income countries to incorporate political economy and policy analysis perspectives within their existing doctoral theses. The programme includes intensive training and technical support to these students from global leaders in the field, coupled with financial assistance to carry out political economy research. Articles reporting research studies supported by the first round of the programme have been compiled into a single publication.^15^

## Lessons learnt

Rigorously incorporating political economy analysis into technical work enables participatory approaches that enhance representation and balance power; improves the feasibility of adopting and implementing technically sound policies; and builds capacity to develop sensitive and balanced policy approaches (Box 2). Explicitly recognizing and managing underlying political and economic power structures strengthens the quality of our technical work. This approach can also help us to distinguish science from political and social processes, including the way in which that science is interpreted and incorporated into health policies.

Box 2 **Summary of main lessons learnt**

- Political economy analysis enables participatory approaches that enhance representation and balance power dynamics.
- Incorporating political economy analysis helps to improve the adoption and implementation feasibility of technically sound health policies.
- Building capacity in using political economy analysis promotes the development of sensitive and balanced health policy approaches.

Each of the workstreams we outline above seeks to build capacity to systematically understand and incorporate political economy dynamics and factors into our technical work to support improved health. In the examples provided, political economy analysis does not supplant technical analysis or guidance, but rather complements it. In doing so, we hope to begin a process whereby political economy analysis can be integrated into technical analysis, policies and interventions to improve health, rather than viewed as something separate and distinct. Given the inherently political nature of public health, this process is not straightforward – whether at the global, national or subnational level. With these examples we seek to clarify political economy analysis and encourage its use as part of technical work to advance UHC and health for all.
